# Perforated Meckel's Diverticulum in an Adult that Resembles Acute Appendicitis: A Case Report and Review of Literature

**DOI:** 10.2174/0115734056320557241211115403

**Published:** 2025-01-02

**Authors:** Noha Bakhsh, Mai Banjar

**Affiliations:** 1 Department of Internal Medicine, Division of Radiology, Faculty of Medicine, King Abdulaziz University, Rabigh, Saudi Arabia; 2 Medical Imaging Department, King Abdullah Medical Complex, Jeddah, Saudi Arabia

**Keywords:** Meckel's diverticulum (MD), Adult, Perforation, Case report, Imaging, ultrasound

## Abstract

**Background:**

Perforation is one of the rarest effects of Meckel's diverticulum and may clinically resemble acute appendicitis.

**Case Report:**

A 34-year-old woman with pain in the right iliac fossa, nausea, and vomiting for three days was brought to the emergency department. An abdominal examination indicated rebound tenderness in the area of the right iliac fossa. Abdominal ultrasound showed a heterogeneous lesion in the left iliac fossa measuring 5 cm × 3.5 cm × 4 cm with no internal vascularity. Abdominal Computed Tomography (CT) demonstrated a hypodense lesion located left of the midline of the abdomen, which was inseparable from the small bowel at the antimesenteric border. Laparoscopic exploration was performed, and an intraoperative diagnosis of perforated Meckel’s diverticulum with phlegmon formation was made. The patient had an uneventful recovery.

**Conclusion:**

Radiologists should be aware of the possibility of complicated Merkel's diverticulum when encountering cases of acute abdominal pain. If there is a lower abdominal inflammatory process and a normal appendix is identified, there should be a high degree of suspicion when examining the CT scan.

## 1 INTRODUCTION

The most common congenital gastrointestinal tract malformation is Meckel's Diverticulum (MD), which affects 2% of the population and poses a 4.2–6.4% risk of problems [1]. German anatomist John Meckel first reported the condition in 1809 [2]. This condition is described using the well-known “rule of 2s”, which refers to its 2% occurrence, its distance of 2 feet away from the ileocecal valve, its length of 2 inches, involvement of one or two kinds of heterotopic pancreatic or stomach tissue, and typical symptoms occurring by the time a child is 2 years old [3].

Children are more likely to have MD symptoms, but adults may also experience complications, including bleeding in 11.8% of cases, intestinal obstruction in 36.5%, inflammation in 12.7%, intussusception in 13.7%, and neoplasm in 3.2%. Perforation has been found to be the cause of 0.5% of symptomatic diverticulum cases, although it is extremely uncommon [4-6]. Asymptomatic MD has no known sex propensity, but males are more likely than females to experience symptomatic MD [7].

## 2 CASE PRESENTATION

A 34-year-old woman with pain in the right iliac fossa, nausea, and vomiting for three days, but no fever was brought to the emergency department. She had undergone a left salpingectomy 8 years prior due to an ectopic pregnancy. The patient was conscious, alert, and oriented during the physical examination. An abdominal examination indicated rebound tenderness in the area of the right iliac fossa. Her blood pressure was 97/51 mmHg, her body temperature was 37.2°C, her heart rate was 98 beats per minute, her respiratory rate was 17 breaths per minute, and her oxygen saturation was 98%. The provisional diagnosis in the emergency room was appendicitis *versus* ovarian pathology.

**Fig. (1):**
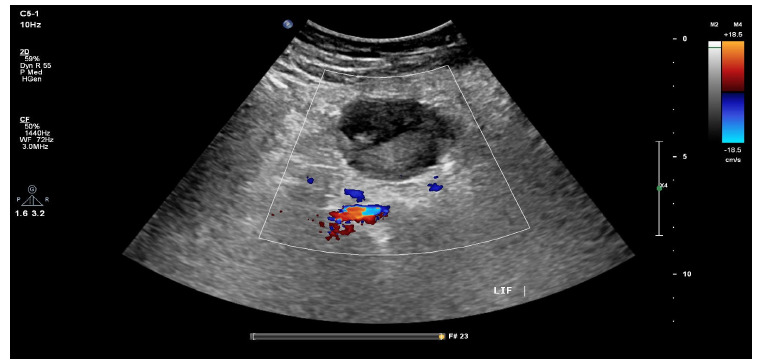
Ultrasound of the left iliac fossa demonstrating a heterogenous lesion measuring 5 cm × 3.5 cm × 4 cm with no internal vascularity.

Fluid replacement and analgesia were first administered to the patient. The patient's laboratory results revealed a platelet count of 298 × 10^3/µl, Hemoglobin level (Hb) of 11.7 g/dl, white blood cell count of 20.0 × 10^3/µl (neutrophil count 17.5 × 10^3/µl, 87.6%), creatinine level of 59.0 µmol/L, and Blood Urea Nitrogen (BUN) of 3.3 µmol/L. X-rays of the patient's abdomen and chest showed nothing unusual. Ultrasound (US) of the abdomen and pelvis revealed normal ovaries with preserved vascularity and minimal pelvic fluid. Evaluation of the right iliac fossa demonstrated non-visualization of the appendix; however, there were no secondary signs to suggest inflammation. There was a heterogenous lesion measuring 5 cm × 3.5 cm × 4 cm in the left iliac fossa with no internal vascularity. Considering the patient's ongoing pain, lack of appendix visualization, and the existence of a poorly characterized structure in the left iliac fossa that could be related to the patient's complaint, an inflammatory appendix in an unusual location should be excluded. Further evaluation with CT, however, is required (Fig. **1**).

**Fig. (2):**
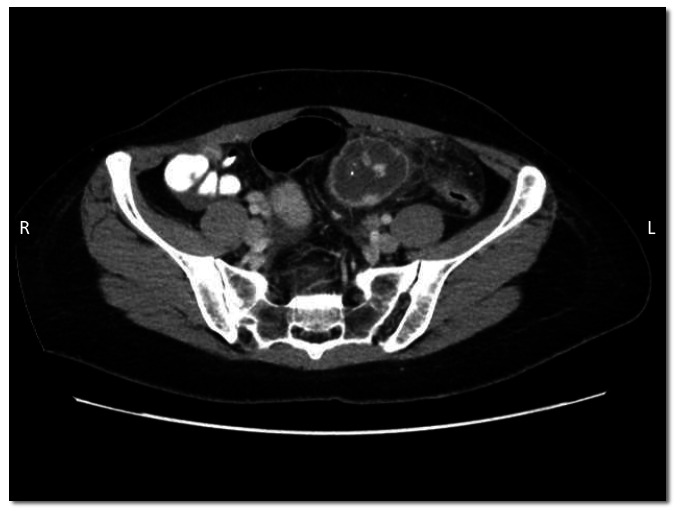
Axial-plane CT of the abdomen and pelvis demonstrating a hypodense lesion located left of the midline of the abdomen containing high-density material suggestive of hemorrhage. This lesion appeared inseparable from the small bowel and was surrounded by inflammatory changes. The adjacent colon showed mild thickening.

**Fig. (3):**
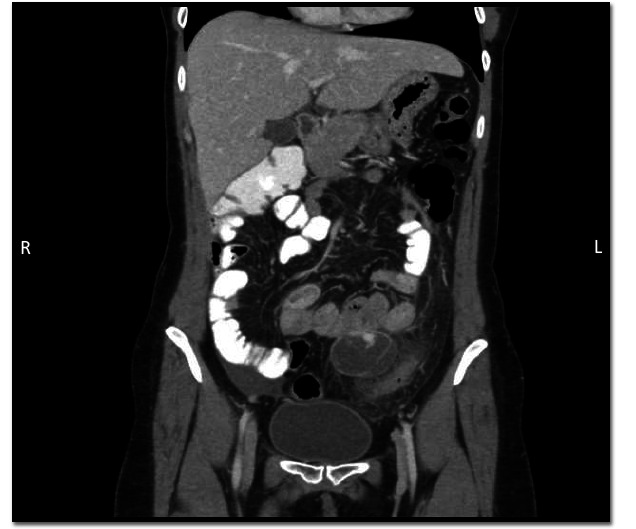
Coronal-plane CT of abdomen and pelvis demonstrating a hypodense lesion located left of the midline of the abdomen containing high-density material suggestive of hemorrhage. This lesion appeared inseparable from the small bowel and was surrounded by inflammatory changes. The adjacent colon showed mild thickening.

The patient was admitted for general surgery for acute appendicitis, given Intravenous (IV) antibiotics, and received nothing by mouth to prepare for possible surgery. Using IV and oral contrast, a Computed Tomography (CT) scan of the abdomen and pelvis showed a modest amount of free fluid in the pelvis and a normal-looking appendix. A hypodense lesion that corresponded to ultrasound findings in the left of the midline of the abdomen was identified, containing high-density material suggestive of hemorrhage, which was inseparable from the small bowel at the antimesenteric border and had surrounding fat stranding. A mild thickening of the adjacent colon was noted (Figs. **2** and **3**).

Next, laparoscopic exploration was performed. The appendix was identified and resected, which looked grossly normal. A mass with omental adhesion was found near the sigmoid colon at the left abdomen, and resection and anastomosis were performed. An intraoperative diagnosis of perforated MD with phlegmon formation was made. The surgical specimen's histopathology results verified the finding of MD, which was accompanied by severe active chronic inflammation and ulceration with hemorrhage and a non-inflamed appendix. The patient had an uneventful recovery.

## 3 DISCUSSION

MD perforation is a dangerous and frequently fatal complication that typically results from gangrene, diverticulitis, or peptic ulcers due to an ectopic stomach mucosa [8]. It is a rare consequence that resembles acute appendicitis, but pain usually occurs in the periumbilical area instead of the right iliac fossa [9, 10]. Bennett *et al*. examined the largest series and reported CT findings from 11 patients with symptomatic MD [11]. The diverticulum was situated to the left of the midline in only one patient. In our case, the patient presented with pain in the right iliac fossa away from the site of the diverticulum in the left abdomen, which was confirmed intraoperatively.

Due to its non-specificity in clinical and radiological diagnosis, many cases of MD are discovered after a laparotomy [9]. In CT, it appears as a fluid- or air-filled blind-ending pouch that protrudes from the antimesenteric side of the distal ileum [12, 13]. However, because CT may still show the appearance of a typical bowel loop, it has limited sensitivity for the identification of simple MD [12, 13]. Complicated MD typically results in acute abdominal pain, and most cases of inflamed MD can be observed in CT [12, 13].

MD is deemed complicated when intestinal obstruction occurs at the location of the diverticulum or it exhibits surrounding features of inflammation or infiltration, signs of a perforation, active bleeding, or nearby fluid collection. The diverticulum may present as a loculated fluid or air-fluid collection with normal small bowel proximal and distal to the diverticular inflammation, or it may have surrounding mesenteric inflammatory changes depending on the severity [7]. Preoperative diagnosis of symptomatic MD is made in less than 10% of cases [5]. Alternative techniques, such as routine radiography, are typically ineffective and non-revealing, although they may show signs of intestinal obstruction, enteroliths, and a diverticulum's gas-fluid level [14]. Sonography has been used to investigate MD with limited success [15].

A fluid-filled structure is usually visible in the right lower quadrant on high-resolution sonography that resembles a thick-walled, blind-ending bowel loop, which is linked to a normal peristaltic small bowel loop and has the typical gut signal [1]. In general, technetium-99 radionuclide scanning exhibits more potential as a diagnostic method for MD in pediatric patients and shows 97% sensitivity and 94% specificity. However, because there is no gastric mucosa in adulthood, the percentage drops to 46% in adult cases [16]. Surgical resection is the primary course of treatment for symptomatic MD, and the most often used techniques are diverticulectomy, segmental bowel resection, anastomosis, and wedge resection [17]. The reviewed cases are summarized in Table **1** [9, 18-28].

## CONCLUSION

Radiologists should be aware of the possibility of complicated MD when encountering cases of acute abdominal pain. If there is a lower abdominal inflammatory process and a normal appendix is identified, there should be a high degree of suspicion when examining the CT scan.

## Figures and Tables

**Table 1:** A summary of Meckel’s diverticulum cases reported in the literature.

**Refs.**	Date of Study	Country	Age and Sex	Clinical Presentation	Preoperative Clinical Diagnosis	Preoperative Radiological Diagnosis	Operative Findings	Histopathology Findings
**Farah *et al*. [** **9** **]**	2014	Morocco	26Y M	Right iliac fossa pain for 5 days associated with high-grade fever	Appendicular peritonitis	-	The appendix was normal. Inflamed and perforated Meckel's diverticulum at its base was found 75 cm proximal to the ileocecal valve	Heterotopic mucosa of diverticulitis
**Modi *et al*. [** **18** **]**	2015	Australia	48Y F	Epigastric and periumbilical lower abdomen pain accompanied by anorexia, vomiting, and nausea	Gynecological *versus* intestinal origin of the pain	CT: MD, features of peritonitis, containing calcification	Generalized peritonitis and a perforated Meckel's diverticulum	Perforated MD caused by fecolith without ectopic tissue in MD being detected
**Rafe *et al*. [** **19** **]**	2015	India	19Y M	Abdominal pain in the right lower quadrant accompanied by nausea and fever	Perforated appendix with peritonitis	X-ray: unremarkable US: moderate free fluid with internal echoes and free air in the abdomen CT: moderate amount of free fluid in the pelvis and free air. An enhanced collection was seen in the patient's midline surrounded by inflammatory changes	Normal appearing appendix. Fecolith-containing inflamed and perforated Meckel's diverticulum at the tip	Severe acute inflammation of the small bowel next to a perforated Meckel's diverticulum. No gastric- or pancreatic-type mucosa
**Abizeid *et al*. [** **20** **]**	2017	Saudi Arabia	17Y M	Pain in the lower abdomen accompanied by nausea and vomiting	-	X-ray: unremarkable CT: pockets of intraperitoneal air, and indications of unusual ileal loop thickening and the potential for an MD. Normal appearing appendix and moderate ascites	Big, sessile base MD 1 m from the ileocecal valve on the antimesenteric border. It was inflamed and had a perforation at its tip. The appendix was normal	Heterotopic gastric and pancreatic tissues with diverticulitis and no evidence of malignancy
**Camelo *et al*. [** **21** **]**	2018	Portugal	49Y M	Right abdominal pain	-	X-ray and US: no signs of appendicitis nor any notable changes CT: a perforated MD. Unremarkable appendix	Perforated MD along with extensive inflammation mass (abscess)	Perforated Meckel's diverticulum at its distal portion with contagious abscess. No ectopic tissue
**Fraser *et al*. [** **22** **]**	2018	USA	54Y F	1^st^ admission: abdominal pain in the lower right quadrant accompanied by nausea and fever	Acute appendicitis	CT: no acute abdominal abnormalities, and the appendix appeared normal	Acutely inflamed appendix that seemed to be surrounded by phlegm and extensive inflammatory adhesions in the RLQ. The appendix was removed	Acute serositis of the appendix with no signs of perforation and an uncommon tiny mucosal inflammation
				2^nd^ admission: persistent abdominal pain, worsening symptoms, associated with nausea, fever, and constipation. JP drainage became more pronounced and feculent	Bowel perforation *versus* appendiceal stump dehiscence	-	Enteric contents with significant inflammation throughout. Stapled line dehiscence was not evident. An ileal diverticulum with perforation at its apex was found	Diverticulum featuring perforation and pressure necrosis at the apex. No indications of malignancy or ectopic tissue
**Al Qahtani *et al*. [** **23** **]**	2018	Qatar	26Y F	Right iliac pain associated with nausea and anorexia	Acute appendicitis	US: a small fluid collection in the right iliac area and a picture of perforated acute appendicitis	Perforated narrow-based diverticulum originating from the ileum's mesenteric border. No peritoneal collection, and the appendix appeared healthy	A diverticular pouch with inflammation
**San Martine *et al*. [** **24** **]**	2019	Spain	34Y M	1^st^ admission: nausea and vomiting along with abdominal pain in the right iliac region and hypogastrium	-	US: ileal region inflammation and free fluid CT: ileitis excluding appendix-related illnesses	-	-
				2^nd^admission: increased intensity of diffuse abdominal pain and abdominal distention	-	X-ray: pneumoperitoneum CT: pneumoperitoneum in the right iliac region and hypogastrium, closely linked to ileal bowel loops having inflammatory changes that were consistent with acute perforated Meckel's diverticulitis	Meckel's diverticulitis presenting with localized inflammation and subsequent peritonitis	A diverticular with a perforated region of the mucosa and gastric-appearing mucosa
**Shimagaki *et al*. [** **25** **]**	2020	Japan	16Y M	Right abdominal (hypochondrium) pain	-	X-ray: calcification in the right abdomen. CT: calcification in the extended intestine in the lower abdomen. Peritonitis due to appendicitis or Meckel’s diverticulitis with enterolith and ascites	Perforated MD containing the enterolith was identified, its base was stenotic and perforation was observed in the vicinity. The appendix showed mild inflammation.	A narrow-necked diverticulum containing one enterolith with severe mucosal inflammation and hemorrhage with no ectopic gastric mucosa.
**AlShareef *et al*. [** **26** **]**	2021	Saudi Arabia	70Y M	Left groin swelling	Strangulated inguinal hernia	X-ray: unremarkable CT: unremarkable	2 Meckel’s diverticula: one on the mesenteric side, which was perforated and communicating with the left inguinal opening, and the other on the antimesenteric side	Perforated Meckel's diverticulum associated with dense inflammation and ischemia
**Tupputi *et al*. [** **27** **]**	2021	Italy	23Y M	Abdominal pain	Acute appendicitis *versus* Meckel’s diverticulum inflammation	X-ray: unremarkable CT: blind-ended loop in the right quadrants of the abdomen associated with mesenteric edema and adjacent nodes. Presence of gas nuclei diagnostic of perforation	Meckel’s diverticulitis	-
**Ahmgb *et al*. [** **28** **]**	2022	Srilanka	30Y F	Right lower abdominal pain	-	X-ray: no significant abnormality US: a sizable amount of free fluid in the pelvic and between the intestinal loops, raising the possibility of an appendix perforation	Free bowel material in the abdomen and a perforated Meckel's diverticulum (tip)	Meckel’s diverticulum predominantly lined by small intestinal-type mucosa and ectopic gastric mucosa. There was localized mucosal ulceration and suppurative inflammation of the wall

## Data Availability

All data generated or analyzed during this study are included in this published article.

## LIST OF ABBREVIATIONS

CT: Computed Tomography
MD: Meckel's Diverticulum
Hb: Hemoglobin level
IV: Intravenous

## STANDARDS OF REPORTING

CARE guidelines were followed.
